# Sulforaphane Suppresses Hepatitis C Virus Replication by Up-Regulating Heme Oxygenase-1 Expression through PI3K/Nrf2 Pathway

**DOI:** 10.1371/journal.pone.0152236

**Published:** 2016-03-29

**Authors:** Jung-Sheng Yu, Wei-Chun Chen, Chin-Kai Tseng, Chun-Kuang Lin, Yao-Chin Hsu, Yen-Hsu Chen, Jin-Ching Lee

**Affiliations:** 1 Department of Chinese Medicine, Chi Mei Medical Center, Tainan, 71004, Taiwan; 2 Graduate Institute of Integrated Medicine, China Medical University, Taichung, 40402, Taiwan; 3 Graduate Institute of Medicine, College of Medicine, Kaohsiung Medical University, Kaohsiung, Taiwan; 4 Institute of Basic Medical Sciences, College of Medicine, National Cheng Kung University, Tainan, Taiwan; 5 Center of Infectious Disease and Signaling Research, College of Medicine, National Cheng Kung University, Tainan, Taiwan; 6 Doctoral Degree Program in Marine Biotechnology, College of Marine Sciences, National Sun Yat-Sen University, Kaohsiung, Taiwan; 7 Division of Infectious Diseases, Department of Internal Medicine, Kaohsiung Medical University Hospital, Kaohsiung, Taiwan; 8 School of Medicine, Graduate Institute of Medicine, Sepsis Research Center, Center for Dengue Fever Control and Research, Kaohsiung Medical University, Kaohsiung, Taiwan; 9 Department of Biological Science and Technology, College of Biological Science and Technology, National Chiao Tung University, HsinChu, Taiwan; 10 Center for Infectious Disease and Cancer Research, Kaohsiung Medical University, Kaohsiung, Taiwan; 11 Department of Biotechnology, College of Life Science, Kaohsiung Medical University, Kaohsiung, Taiwan; 12 Graduate Institute of Natural Products, College of Pharmacy, Kaohsiung Medical University, Kaohsiung, Taiwan; 13 Research Center for Natural Products and Drug Development, Kaohsiung Medical University, Kaohsiung, Taiwan; University of Washington, UNITED STATES

## Abstract

Hepatitis C virus (HCV) infection-induced oxidative stress is a major risk factor for the development of HCV-associated liver disease. Sulforaphane (SFN) is an antioxidant phytocompound that acts against cellular oxidative stress and tumorigenesis. However, there is little known about its anti-viral activity. In this study, we demonstrated that SFN significantly suppressed HCV protein and RNA levels in HCV replicon cells and infectious system, with an IC_50_ value of 5.7 ± 0.2 μM. Moreover, combination of SFN with anti-viral drugs displayed synergistic effects in the suppression of HCV replication. In addition, we found nuclear factor erythroid 2-related factor 2 (Nrf2)/HO-1 induction in response to SFN and determined the signaling pathways involved in this process, including inhibition of NS3 protease activity and induction of IFN response. In contrast, the anti-viral activities were attenuated by knockdown of HO-1 with specific inhibitor (SnPP) and shRNA, suggesting that anti-HCV activity of SFN is dependent on HO-1 expression. Otherwise, SFN stimulated the phosphorylation of phosphoinositide 3-kinase (PI3K) leading Nrf2-mediated HO-1 expression against HCV replication. Overall, our results indicated that HO-1 is essential in SFN-mediated anti-HCV activity and provide new insights in the molecular mechanism of SFN in HCV replication.

## Introduction

Approximately 3% of the world’s population is infected by hepatitis C virus (HCV), a major and crucial global health problem [1]. Majority of the infected individuals fail to clear the virus and are at risk of developing crucial liver complications such as cirrhosis and hepatocellular carcinoma (HCC). During the last decade, the standard therapy against hepatitis C was based on combination of ribavirin and pegylated interferon-α (Peg-IFN-α). This treatment showed moderate efficiency against HCV genotype 1-infected patients [2]. Recent progress allowed introducing new antivirals with high anti-HCV activities against different HCV genotypes. An example, Harvoni (sofosbuvir and ledipasvir), recently approved by US Food and Drug Administration (FDA), has shown a significant antiviral activity against different HCV genotypes [3]. Although used in some countries, the currently approved drugs are still limited by their high cost and some side effects. More effective and better-tolerated agents are still needed to reinforce the therapeutic arsenal. Thus, novel anti-HCV agents and therapeutics may improve the new treatment strategies against HCV infection and HCV-associated liver disease.

Sulforaphane (SFN), an isothiocyanate abundant in cruciferous vegetables, is proved to be a cytoprotectant by numerous *in vivo* and *in vitro* studies because of its anti-inflammatory and anti-cancer activities during multiple stages in tumorigenesis [4, 5]. In addition, SFN exhibits a significant antiviral activity against influenza virus, human immunodeficiency virus (HIV), and Epstein-Barr virus [6, 7]. The hepatoprotective effects of SFN are examined based on its antioxidant effects by the concomitant upregulation of the phase II detoxification enzyme expression and downregulation of the phase I detoxification enzyme expression. Furthermore, SFN significantly induces antioxidant response element (ARE)-regulated enzymes, providing a defense against oxidative stress [8]. ARE promoter activity is primarily modulated by BTB and CNC homolog 1 (Bach1) as well as nuclear factor erythroid-derived 2-related factor 2 (Nrf2) that is suppressed by binding to Kelch-like ECH-associated protein 1 (Keap1) [9]. SFN is accordingly suggested to function effectively in regulating ARE promoter activity with the consequent induction of several reactive oxygen species (ROS)-scavenging molecules, including heme oxygenase-1 (HO-1), and to be beneficial in alleviating the risk of oxidative stress-related diseases [10, 11].

Previous studies have shown a significant correlation between HCV replication and cellular oxidative stress, and treatment with antioxidants is considered as a potentially new therapeutic approach for HCV infection [12, 13]. A protective enzymes against oxidative stress, HO-1, catalyzes the degradation of cytotoxic heme into biliverdin, carbon monoxide, and ferrous iron, which are the three major elements in providing cytoprotection. In previous studies, HO-1 induction is shown to interfere with the replication of various viruses such as human immunodeficiency virus and hepatitis B virus [14, 15]. In addition, HO-1 is considered as a potential therapeutic target in HCV therapy. Biliverdin, a product of HO-1-mediated heme catalysis, is demonstrated to be an anti-HCV factor by increasing the antiviral IFN response and inhibiting the HCV NS3/4A protease activity [16, 17].

Here we assessed the anti-HCV activity of SFN and its analogs and demonstrated that SFN significantly inhibited HCV replication. As a potential phytocompound with antioxidant and antiviral properties, SFN may offer an effective therapeutic strategy against HCV-associated liver disease by simultaneously reducing viral infection and chronic inflammation.

## Materials and Methods

### Cell Lines and Reagents

Huh-7, Huh7.5, Ava5 (harboring HCV subgenomic replicon; genotype 1b) and Huh7.5/J6/JFHEMCVIRESRlucNeo cells (harboring HCV subgenomic replicon RNA and renilla luciferase reporter gene; genotype 2a) obtained from Apath, LLC (St. Louis, MO) [18] were routinely passaged in Dulbecco’s modified Eagle’s medium containing 10% heat-inactivated fetal bovine serum, 1% nonessential amino acids, and 1% antibiotic-antimycotic in a 37°C incubator with a humidified atmosphere containing 5% CO_2_. Sulforaphane (SFN), phenethyl isothiocyanate, benzyl isothiocyanate, benzyl isothiocyanate, butyl isothiocyanate, allyl isothiocyanate, and HO-1-specific inhibitor (tin protoporphyrin IX dichloride; SnPP) were obtained from Sigma Aldrich Co. (St. Louis, MO, USA). Biliverdin was obtained from MP Biomedicals, Inc (Santa Ana, CA, USA). IFN-α-2a (Roferon-A) was obtained from Roche Ltd (Basel, Switzerland). Daclatasvir and telaprevir were obtained from Legend Stat International Co., Ltd (Omdurman, Sudan). Sofosbuvir was obtained from Shanghai Haoyuan Chemexpress Co., Ltd. (Shanghai, China). These compounds were stored at a concentration of 10 mM in 100% dimethyl sulfoxide (DMSO) at 4°C until use. All the reactions were performed using a final concentration of 0.1% DMSO.

### HCV JFH-1 Infection

HCV infectious particles (JFH-1; genotype 2a) were produced as previously described [19]. HCV infection assay was used to determine the antiviral effect of SFN. Huh-7 cells were seeded in 24-well plates at a density of 5 × 10^4^ cells per well and were infected with HCV for 6 h at a multiplicity of infection (MOI) of 0.1. The HCV-infected cells were treated with different concentrations (0–10μM) of SFN and incubated for 3 days, following which total RNA samples were extracted and subjected to qRT-PCR analysis for the quantification of HCV replication.

### Synergistic Effects of SFN and Antiviral Drugs on Infectious and Replicon Systems

HCV-infected and Ava5 cells were treated with serial concentrations of SFN (0, 1.25, 2.5, 5.0, 7.5 and 10.0 μM) combined respectively with or without IFN-α (7.5, 15, 30, and 60 U·mL^-1^), telaprevir (0.075, 0.15, 0.3, and 0.6 μM), sofosbuvir (10, 20, 40, and 80 nM) or daclatasvir (1, 2, 4, and 8 pM) in a fixed ratio for 3 days. The anti-HCV activities of the different combinations were determined by qRT-PCR. The drug combination data were analyzed using CalcuSyn2™ software (Biosoft, Cambridge, UK) based on the method by Chou and Talalay [20]. The multiple drug dose effects and combination index (CI) values were evaluated and presented as antagonism (CI > 1), additivity (CI = 1) or synergism (CI < 1).

### Quantitative Real-Time PCR and Reverse Transcription

Total cellular RNA extracted from cultured cells was purified using the Total RNA Miniprep Purification Kit (GMbiolab Co., Ltd, Taiwan). RNA samples were reverse transcribed into complementary DNA using the M-MLV Reverse Transcription System (Promega). Quantitative real-time polymerase chain reaction (qRT-PCR) was performed to assess the relative levels of gene expression using the ABI Step One Real-Time PCR-System (ABI Warrington, UK). Glyceraldehydes-3-phosphate dehydrogenase (*gapdh*) gene expression was used as the endogenous control for normalization in all qRT-PCR analyses. Relative expression was calculated using the comparative Ct method. Absolute quantitation of HCV RNA copies was calculated and determined relative to standard curves comprised of serial dilution of expression plasmids containing the coding sequence of HCV NS5B using RT-qPCR. The primer sequences primers for qRT-PCR were used as previously described (Table 1).

**Table 1:** Oligonucleotide sequences for real-time RT-PCR.

Oligonucleotide Name	Sequence 5'-3'
5' NS5B	5'-GGA AACCAAGCTGCCCATCA
3' NS5B	5'-CCTCCACGGATAGAAGTTTA
5' GAPDH	5'-GTCTTCACCACCATGGAGAA
3' GAPDH	5'-ATGGCATGGACTGTGGTCAT
5'OAS1	5’- CAAGCTTAAGAGCCTCATCC
3'OAS1	5’- TGGGCTGTGTTGAAATGTGT
5'OAS2	5’- ACAGCTGAAAGCCTTTTGGA
3'OAS2	5’- GCATTAAAGGCAGGA AGCAC
5'OAS3	5’- CACTGACATCCCAGACGATG
3'OAS3	5’- GATCAGGCTCTTCAGCTTGG
5'PKR	5’- ATGATGGAAAGCGAACAAGG
3'PKR	5’- GAGATGATGCCATCCCGTAG
5'BVR	5’- ATGAGGGACTTGCGGAAT
3'BVR	5’- ATTAAGGAACTGCCTGATGTAG
5'IFN-α2	5’- GCAAGTCAAGCTGCTCTGTG
3'IFN-α2	5’- GATGGTTTCAGCCTTTTGGA
5'IFN-α17	5'-AGGAGTTTGATGGCAACCAG
3'IFN-α17	5'-CATCAGGGGAGTCTCTTCCA

### Cell Viability

The CellTiter 96 Aqueous One Solution Cell Proliferation assay system (Promega, Madison, WI, USA), which is a colorimetric approach for the detection of the cytotoxic effect of a compound, was used to measure cell viability according to the manufacturer’s protocol. Ava5 cells were seeded in 96-well plates at a density of 5 × 10^3^ cells per well and treated with SFN at indicated concentrations for 3 days. The absorbance values were detected at 490 nm using a 96-well plate reader (Bio-Rad, Hertfordshire, UK).

### Western Blot Assay

Western blotting was performed as previously described [21]. Proteins from cell lysates were separated using SDS-PAGE and transferred onto PVDF membranes. The membranes were blocked with 5% nonfat milk in phosphate-buffered saline containing Tween 20 (PBST) and incubated overnight at 4°C with primary antibodies against GAPDH (1:10,000; GeneTex, CA, USA), NS5B (1:5,000; Abcam, Cambridge, MA, USA), Nrf2 (1:3,000; GeneTex), HO-1 (1:3,000; GeneTex), biliverdin reductase A (BVRA), Keap1 and Bach1 (1:1,000; Abcam), and total-phohphoinositide 3-kinase (PI3K) or phosphor-PI3K (1:1000; Cell Signaling Technology, Inc., Beverly, MA, USA). The immunoreactive blot signals were detected using the enhanced chemiluminescence Western blotting reagent (Perkin-Elmer, CT, USA).

### HCV Entry and Assembly Assay

The effect of SFN on HCV entry was determined according to previously described methods [22]. Briefly, Huh7.5 cells were infected with JFH-1 and treated with SFN for 1 hour at 37°C (endocytosis/fusion period). Finally, cells were washed and incubated in complete culture medium at 37°C for 45 hours. Subsequently, the cells were collected to quantify intracellular HCV RNA copy numbers. The effect of SFN on assembly and secretion were determined according to previously described methods [23]. The Huh7.5 cells were infected with JFH-1 and treated with indicated concentration of SFN. The viral supernatants and cell pellets were collected at 3 days postinfection for virus titration by qRT-PCR.

### Transfection and Reporter Activity Assay

The luciferase reporter plasmids used for transcription activity include pHO-1-Luc, p3xARE-Luc, and pISRE-Luc. pHO-1-Luc contains a human HO-1 promoter driving firefly luciferase expression. p3xARE-Luc, containing three repeats of the Nrf2-dependent antioxidant response element (ARE), was used to detect the translocation and transcription activity of Nrf2. pISRE-Luc, containing an IFN-stimulated response element (ISRE) that controls the firefly luciferase expression, was used to detect the activity of IFN response-dependent transcription. The reporter plasmids were transfected into Ava5 cells using the T-Pro*™* reagent following the manufacturer’s instructions (Ji-Feng Biotechnology Co., Ltd., Taiwan). Following incubation for 3 days, cell lysates were extracted and luciferase activities were analyzed using Bright-Glo Luciferase assay system (Promega). To normalize each transfection efficiency, the cells were co-transfected with 0.1 μg of secreted alkaline phosphatase (SEAP) expression vector (pSEAP). The luciferase activity was normalized according to the SEAP activity. The HO-1 (NM_002133), Nrf2 (NM_006164), and EGFP small hairpin RNAs (shRNAs) were purchased from the National RNAi Core Facility, Institute of Molecular Biology/Genomic Research Center, Academia Sinica, Taiwan. These specific shRNAs were used to confirm the mechanism underlying the SFN-mediated anti-HCV activity. All DNA fragments were confirmed by DNA sequencing.

### Cell-Based NS3/4A Protease Activity Assay

Huh-7 cells were co-transfected with the NS3/4A protease reporter vector pEG(DEΔ4AB)SEAP and NS3/4A expression vector pCMV-NS3/4A-myc followed by incubation with the indicated concentrations of SFN (0–10 μM) with or without SnPP (20 μM) for 3 days. The cells were co-transfected with 0.1 μg of firefly luciferase expression vector (pFLuc) for normalization of each transfection efficiency. The supernatants were harvested for measurement of SEAP activity by Phospha-Light assay kit (Tropix, Bedford, UK).

### Measurement of Intracellular Bilirubin

Intracellular bilirubin level was quantified as previously described [24]. Cells were seeded in 6-well plates at a density of 4 × 10^5^ cells per well and treated with SFN at indicated concentrations for 3 days. Cell lysates were harvested and the amount of bilirubin was quantified using Calibrator for Automated Systems (c.f.a.s) (Roche Diagnostics Ltd., South San Francisco, CA, USA) and MeDiPro direct bilirubin test kit (Formosa Biomedical Technology Corp., Taipei, Taiwan). The absorbance values at 546 and 660 nm were measured using the Epoch microplate spectrophotometer (BioTek Instruments, Inc., Winooski, VT, USA).

### Measurement of Extracellular IFN-α

Extracellular IFN-α level was quantified as previously described [25]. Ava5 cells were seeded in 24-well plates at a density of 4 × 10^4^ cells per well and treated with indicated concentrations of SFN with or without SnPP for 3 days. The supernatant was harvested to quantify the production of IFN-α using Human IFN-α ELISA Kit (USCN Life Science Inc., Wuhan, China) according to the manufacturer’s protocol. Absorbance values at 450 nm were measured using the Epoch microplate spectrophotometer (BioTek Instruments, Inc.).

### Cytoplasmic and Nuclear Protein Extraction

Cytoplasmic and nuclear protein extraction was performed as previously described [24]. In brief, Ava5 cells were treated with indicated concentrations of SFN and were collected at different time points. The cells were lysed using an ice-cold hypotonic buffer and the nuclear pellets were separated with high-salt nuclear extraction buffer. Phosphatase and protease inhibitors were added to the lysis and extraction buffer before used. The cytoplasmic and nuclear proteins were collected and stored at −80°C before used.

### Statistical Analysis

Results of five independent experiments are presented as means ± SD. Student’s *t*-test was used to analyze the statistical differences which performed by GraphPad Prism Software (San Diego, California, USA). The level of significant difference was set at *P < 0.05 or **P < 0.01.

## Results

### SFN Inhibits HCV Replication in the Replicon and Infection Systems

**Fig 1:**
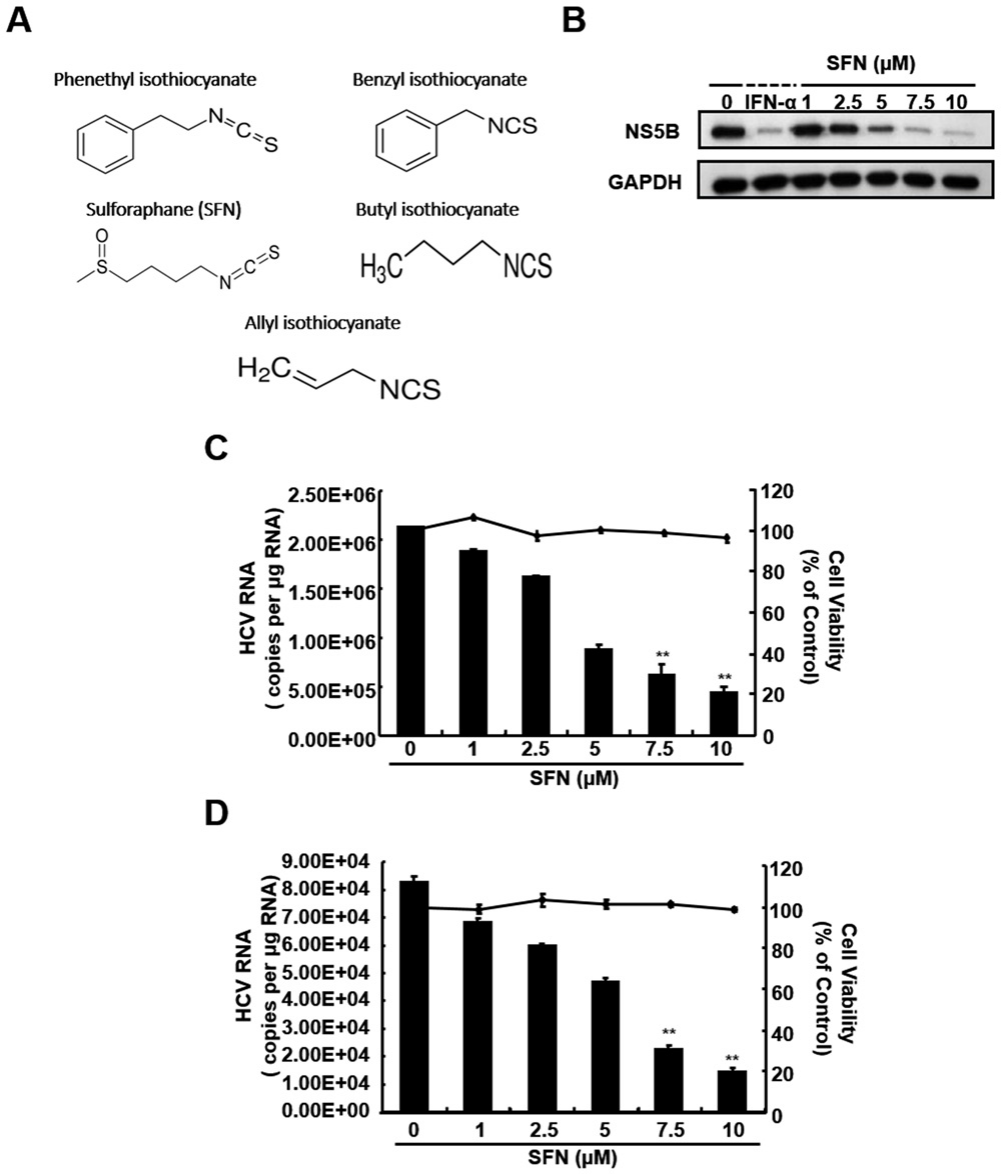
SFN suppressed HCV protein expression and RNA replication. (A) Structures of SFN and its analogs. (B, C) HCV protein and RNA expression were decreased by SFN treatment in the HCV replicon system. Ava5 cells were exposed to the indicated SFN concentrations (0–10 μM) for 3 days. IFN-α at 100 U·ml^−1^ served as the positive control. (D) HCV RNA replication decreased by SFN treatment in the HCV JFH-1 infection system. JFH-1 virus particle-infected Huh-7 cells were exposed to increasing concentrations of SFN for 3 days. Total cellular protein extracts and RNA were determined by Western blotting using specific antibodies and qRT-PCR using primers for NS5B and GAPDH. GAPDH was used as the protein loading control. The absolute HCV RNA copy number was quantified following normalization of the internal control *gapdh*. Viability of Ava5 and Huh-7 cells treated with SFN was determined by the MTS assay. The relative cell viability were presented as percentage changes compared to the SFN-untreated cells, which were considered as 100%. Data were presented as the means of normalized data ± standard deviations (error bars) based on five independent experiments. *P < 0.05; ** P < 0.*01*.

To investigate the anti-HCV activity of SFN and its analogs (Fig 1A), Huh-7-derived Ava5 cells that harbor an HCV subgenomic RNA replicon were treated with increasing concentrations of SFN for 3 days. The anti-HCV activity and cytotoxicity of SFN and its analogs were analyzed by qRT-PCR and MTS assay, respectively. As shown in Table 2, the compound with the best selective index [SI, cytotoxic concentration (CC_50_)/half maximal effective concentration (EC_50_)]among the tested compounds was SFN with an approximate SI value of 10. Moreover, the anti-HCV replication activity of SFN was analyzed by Western blotting and qRT-PCR. The results indicated that SFN significantly reduced HCV protein synthesis and RNA replication in a concentration-dependent manner (Fig 1B and 1C). The calculated EC_50_ of SFN for the reduction of HCV RNA levels was 5 ± 0.5 μM, and an efficient 90% inhibition of viral replication by SFN was observed at a concentration of 10 μM compared to the SFN-untreated cells. The similar results were also found in JFH-1 replicon system (S1 Fig). Assessment of cell viability by MTS assay showed that SFN did not induce significant cytotoxicity at doses where viral replication was inhibited (Fig 1C, right axis), a result that ruled out cytotoxicity as a potential mechanism underlying its antiviral activity. Similarly, we confirmed that SFN exhibited antiviral activity in the HCV JFH-1 infection system (Fig 1D). In addition, there is no significant inhibition on viral entry and assembly by SFN treatment (S2 Fig).

**Table 2:** Anti-HCV activity and cytotoxicity of SFN and its analogs.

Compound Name	Anti-HCV activity (EC_50_)	Cyto- Toxicity (CC_50_)	Selective Index (SI)
**Phenethyl isothiocyanate**	**2.5±0.5 μM**	**10±1.5 μM**	**5**
**Sulforaphane**	**5±1.5 μM**	**52±3 μM**	**10**
**Benzyl isothiocyanate**	**20±2 μM**	**67±2 μM**	**3**
**Butyl isothiocyanate**	**N.D.**	**95±6 μM**	**N.D.**
**Allyl isothiocyanate**	**20±2 μM**	**33±3 μM**	**1.5**

Ava5 cells were treated with various concentrations of SFN or its analogs. The anti-HCV activity and cytotoxicity were analyzed by qRT-PCR and MTS assay, respectively. The relative HCV RNA levels and cell viability were presented as percentage changes compared to untreated Ava5 cells, in which level was considered as 100%. Data were represented as the means of normalized data ± standard deviations (error bars) from five independent experiments. N.D.: not determined.

### SFN in Combination with Antiviral Drugs Exhibits Synergistic Anti-HCV Activity

Newer approaches combining therapeutics simultaneously targeting different viral factors are considered as promising strategies to avoid side effects, control cost, and prevent the emergence of resistant mutants. Therefore, we determined the anti-HCV effects of SFN in combination with each US FDA approved drug, including IFN-α, the NS3/4A protease inhibitor telaprevir, the HCV NS5A inhibitor daclatasvir, and the HCV RNA-dependent RNA polymerase (RdRp) inhibitor sofosbuvir. HCV-infected cells were treated with increasing concentrations of SFN and anti-HCV drugs at a fixed ratio for 3 days, as described in the Materials and methods section. The antiviral effects were analyzed by qRT-PCR, and the synergistic effects of combination treatments were analyzed by the isobologram method using CalcuSyn2^™^ software [20, 26]. As shown in Table 3, CI values at the effective dose of ED_50_, ED_75_, and ED_90_ values ranged from 0.4 to 0.8, indicating that the combination of SFN with antiviral drugs showed a synergistic inhibition of HCV replication. The similar results were also found in replicon system with the CI values ranged from 0.5 to 0.8 (Table 4). These results further confirm that SFN is a potentially important adjuvant that can be incorporated into newer antiviral regimens against HCV.

**Table 3:** Anti-HCV activity of SFN in combination with antiviral drugs in HCV-infected cells.

Combination compound	CI values at			Influence
	ED50	ED75	ED90	
**IFN-α**	**0.8**	**0.8**	**0.4**	**Synergistic**
**Telapriver**	**0.8**	**0.6**	**0.5**	**Synergistic**
**Sofobuvir**	**0.6**	**0.5**	**0.4**	**Synergistic**
**Daclatasvir**	**0.6**	**0.5**	**0.4**	**Synergistic**

JFH-1 virus particle-infected Huh-7 cells were treated with increasing concentrations of SFN in combination with antiviral compounds in a fixed ratio for 3 days as described in the Materials and methods section. The antiviral effects was determined by qRT-PCR, and the isobologram method as used to assess drug–drug interactions. The combination index (CI) values at the effective dose achieving 50% (ED50), 75% (ED75) and 90% (ED90) inhibition were calculated by CalcuSyn2^™^ software and were presented as antagonism (CI > 1), additivity (CI = 1), or synergism (CI < 1).

**Table 4:** Anti-HCV activity of SFN in combination with antiviral drugs in Ava5 cells.

Combination compound	CI values at			Influence
	ED50	ED75	ED90	
**IFN-α**	**0.8**	**0.8**	**0.5**	**Synergistic**
**Telapriver**	**0.8**	**0.7**	**0.6**	**Synergistic**
**Sofobuvir**	**0.6**	**0.5**	**0.4**	**Synergistic**
**Daclatasvir**	**0.6**	**0.5**	**0.4**	**Synergistic**

Ava5 cells were treated with increasing concentrations of SFN in combination with antiviral compounds in a fixed ratio for 3 days as described in the Materials and methods section. The antiviral effects were determined by qRT-PCR, and the isobologram method as used to assess drug–drug interactions. The combination index (CI) values at the effective dose achieving 50% (ED_50_), 75% (ED_75_) and 90% (ED_90_) inhibition were calculated by CalcuSyn2^™^ software and were presented as antagonism (CI > 1), additivity (CI = 1), or synergism (CI < 1).

### SFN Induces HO-1 Expression in HCV Replicon System

**Fig 2:**
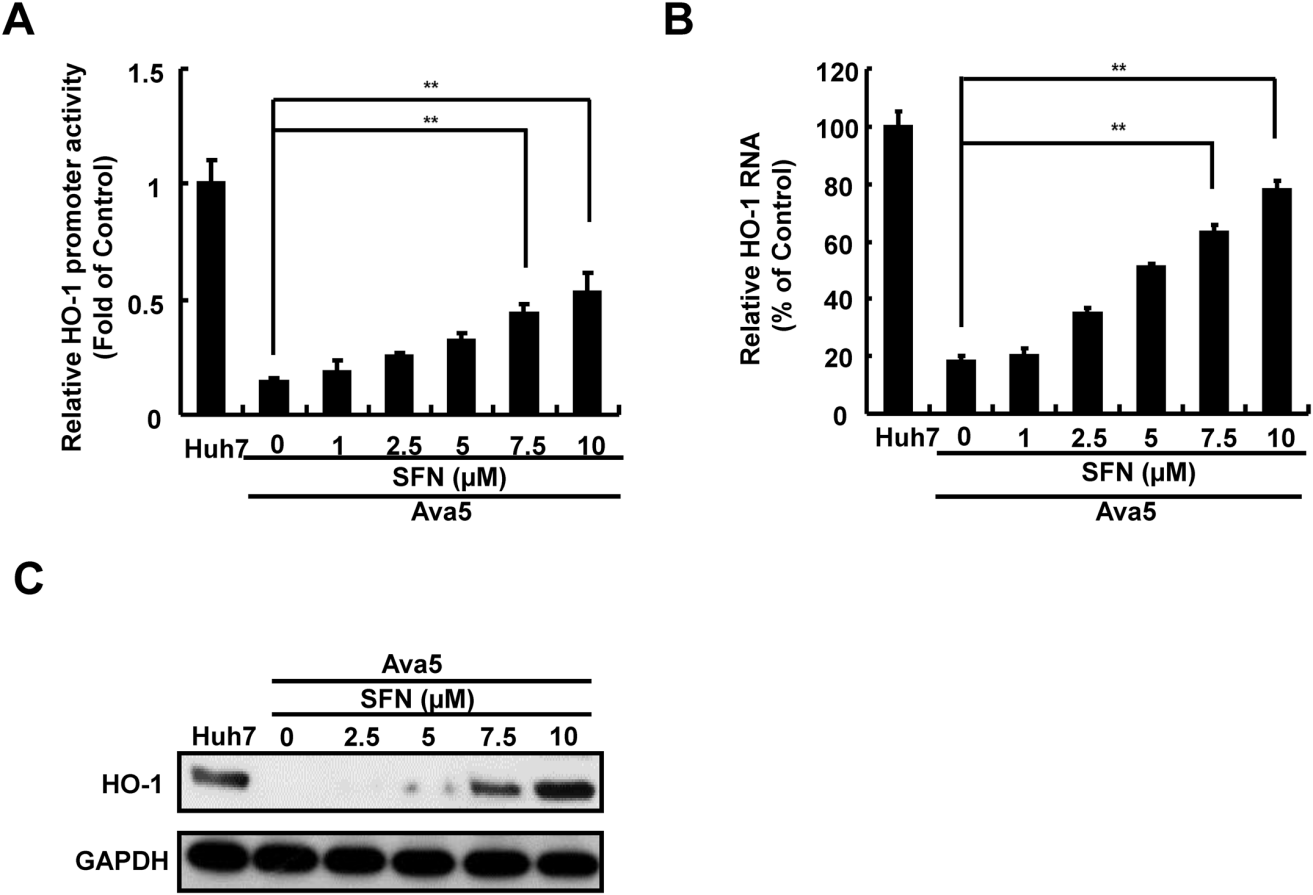
SFN-mediated HO-1 induction in HCV replicon cells. (A) HO-1 promoter activity was assessed in SFN-treated HCV replicon cells. The HO-1 promoter reporter vector, pHO-1-Luc, was transfected into Huh-7 and Ava5 cells. Subsequently, the transfected cells were exposed to the indicated concentrations of SFN for 3 days. The cell lysates were collected, and the luciferase activity was assessed to determine HO-1 promoter activity. (B, C) HO-1 mRNA and protein expression were induced by SFN treatment in the HCV replicon system. Ava5 cell were treated with the indicated concentrations (0–10 μM) of SFN and analyzed by qRT-PCR and Western blotting using specific primers and antibodies for HO-1 and GAPDH, respectively. GAPDH expression was used as the protein loading control. Means of relative RNA levels were normalized to the internal control, *gapdh*. The relative HO-1 promoter activity and HO-1 RNA levels were presented as fold changes compared to parental Huh-7 cells, in which the activity and level were considered as 1 and 100%, respectively. Data were represented as the means of normalized data ± standard deviations (error bars) from five independent experiments. *P < 0.05; ** P < 0.*01*.

HO-1 is considered as a potential therapeutic target against oxidative stress, while its product biliverdin is shown to markedly reduce HCV replication through the induction of antiviral IFN responses and inhibition of HCV protease activity [16, 17]. More recently, SFN is shown to downregulate ROS by inducing the endogenous Nrf2/HO-1 antioxidant response [27]. To determine whether SFN could activate HO-1 expression in HCV replicon cells, we performed an HO-1 promoter-based firefly luciferase reporter assay in Ava5 cells. The pHO-1-Luc-transfected Ava5 cells were incubated with increasing concentrations of SFN for 3 days. As shown in Fig 2A, the HO-1 promoter activity was significantly reduced in Ava5 cells compared to parental Huh-7 cells, and the SFN treatment completely suppressed this inhibitory effect compared to SFN-untreated controls. Similarly, we observed that SFN significantly increased HO-1 RNA and protein levels as detected by qRT-PCR and Western blotting, respectively (Fig 2B and 2C).

### SFN Inhibits HCV Replication by Induction of HO-1 Expression

**Fig 3:**
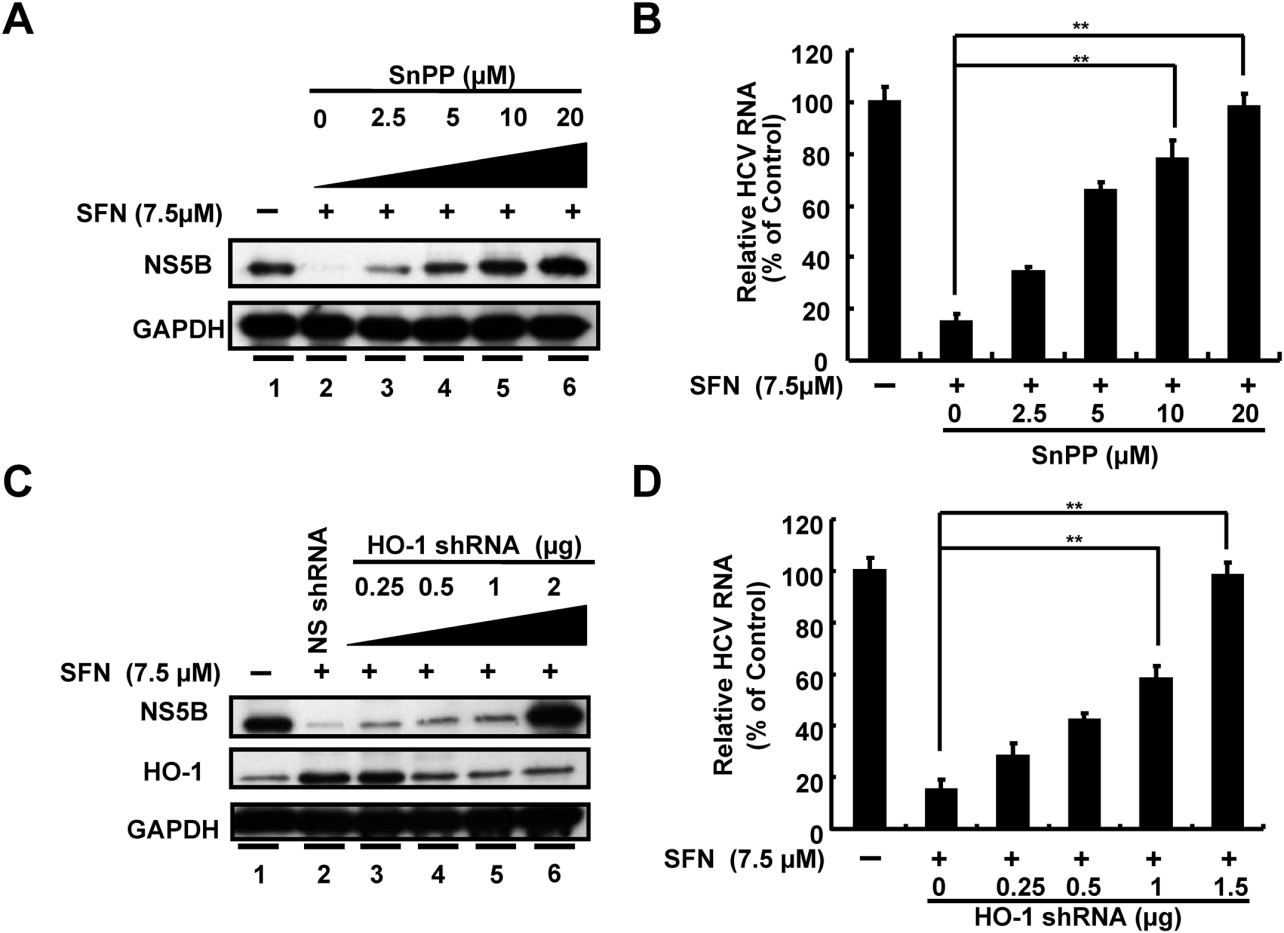
SFN inhibited HCV replication through HO-1 dependent pathway. (A, B) Ava5 cells were treated with increasing concentrations of SnPP (0–20 μM) with or without 7.5 μM SFN for 3 days. HCV protein synthesis and RNA replication were analyzed by Western blotting and qRT-PCR, respectively. (C and D) Increasing amounts of HO-1-specific shRNA (0.25–2 μg) or nonspecific shRNA were transfected into Ava5 cells that were then treated with or without SFN (7.5 μM). After 3 days, HCV protein synthesis and RNA replication were analyzed by Western blotting and qRT-PCR, respectively. Western blotting was performed using antibodies against HCV NS5B and HO-1. An antibody against GAPDH was used to show equal loading of lysates. Relative RNA levels were normalized to the internal control, *gapdh*. The relative HCV RNA levels were presented as percentage changes compared to SFN-untreated/untranfected Ava5 cells, in which level was considered as 100%. Data were represented as the means of normalized data ± standard deviations (error bars) from five independent experiments. *P < 0.05; ** P < 0.*01*.

To further investigate whether the anti-HCV activity of SFN was HO-1-dependent, Ava5 cells were treated with 7.5 μM SFN combined with increasing concentrations of SnPP (0–20 μM) for 3 days. HCV protein synthesis and RNA replication were analyzed by Western blotting and qRT-PCR, respectively. As shown in Fig 3A, the inhibitory effect of SFN on HCV protein synthesis was restored by the SnPP treatment (lanes 3–6) as compared to both no treatment (lane 1) and SFN treatment (lane 2). Similar to these Western blotting results, SnPP treatment significantly attenuated SFN-suppressed HCV RNA levels in a concentration-dependent manner (Fig 3B). To eliminate the potential off-target effects of pharmacological inhibitors, we performed genetic knockdown of HO-1 by shRNA. The specific HO-1 shRNA (0.25–2 μg) or nonspecific control shRNA was transfected into Ava5 cells, and the transfected cells were treated with 7.5 μM SFN for 3 days. HO-1 induction and HCV replication were assessed by Western blotting and qRT-PCR, respectively. Consistent with the abovementioned results observed with SnPP treatment, HO-1 shRNA significantly reduced SFN-mediated HO-1 induction (Fig 3C, middle panel, lanes 3–6), which occurred in parallel with the restoration of HCV protein synthesis (Fig 3C, upper panel, lanes 3–6). HO-1 protein appeared to be inversely correlated with HCV protein synthesis. Similarly, the recovery of HCV RNA levels coincided with decreasing HO-1 protein levels by HO-1 shRNA in SFN-treated cells (Fig 3D). Taken together, these results clearly demonstrated that HO-1 upregulation contributed to the antiviral action of SFN.

### SFN Stimulates Biliverdin Production to Activate Antiviral IFN Response and Suppresses HCV Protease Activity

**Fig 4:**
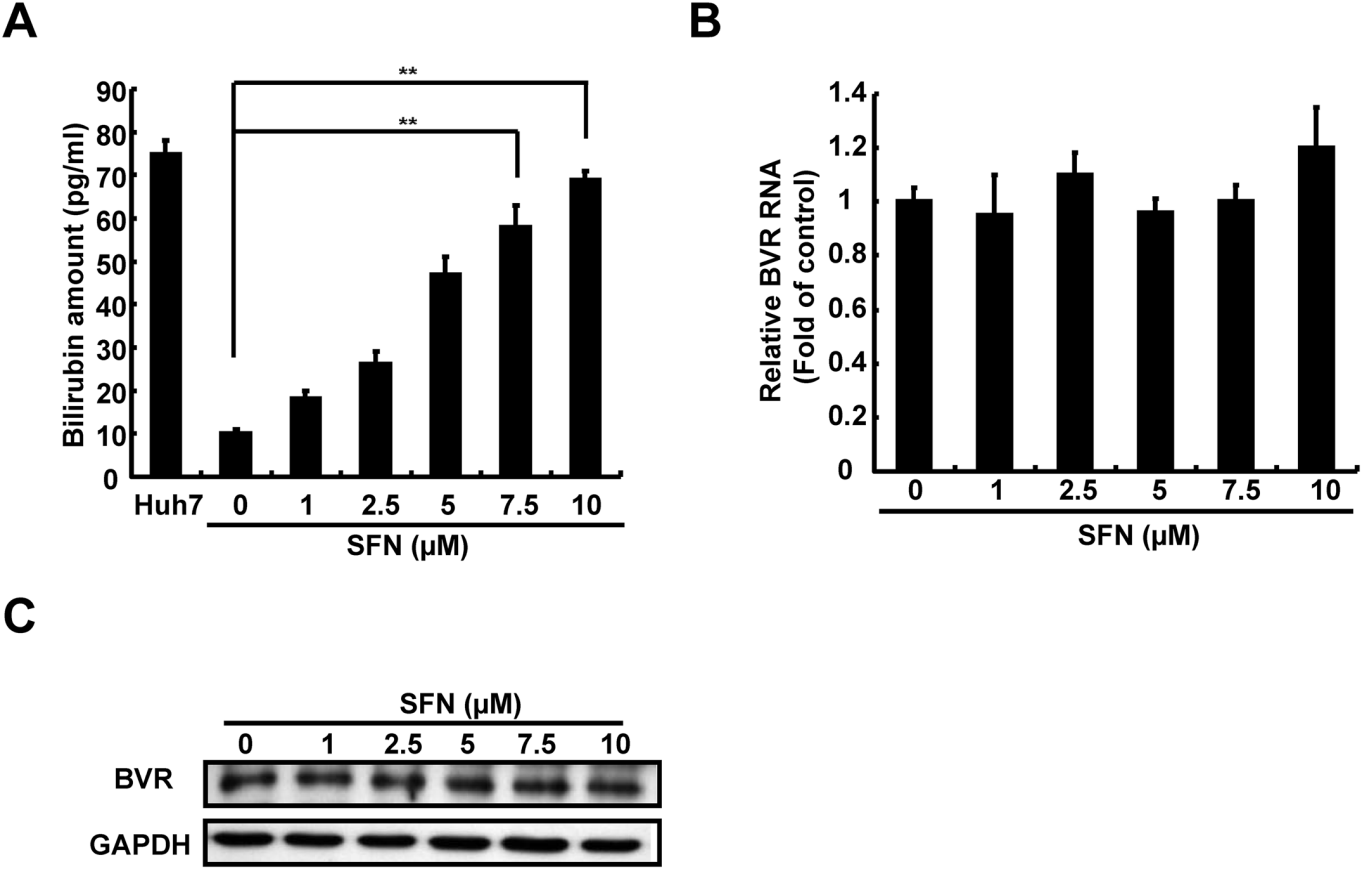
SFN induced bilirubin production in HCV replicon cells. (A) Bilirubin production was induced by SFN treatment in a dose-dependent manner. Ava5 cells were treated with the indicated concentrations of SFN (0–10 μM) for 3 days, and bilirubin level was analyzed by the MeDiPro direct bilirubin test kit and Calibrator for Automated Systems. The parental Huh-7 cells were used to determine basal bilirubin levels. (B and C) Biliverdin reductase A (BVR) mRNA and protein expression did not show significant changes by SFN treatment in the HCV replicon system. Ava5 cells were treated with the indicated concentrations of SFN for 3 days. The expression of BVR was analyzed by qRT-PCR and Western blotting using specific primers and antibodies for BVR and GAPDH, respectively. GAPDH expression was used as protein loading control. Means of relative RNA levels were normalized to the internal control *gapdh*. The relative BVR RNA levels were presented as fold changes compared to parental Huh-7 cells, in which the level was considered to be 1. Data were represented as the means of normalized data ± standard deviations (error bars) based on five independent experiments. *P < 0.05; ** P < 0.*01*.

**Fig 5:**
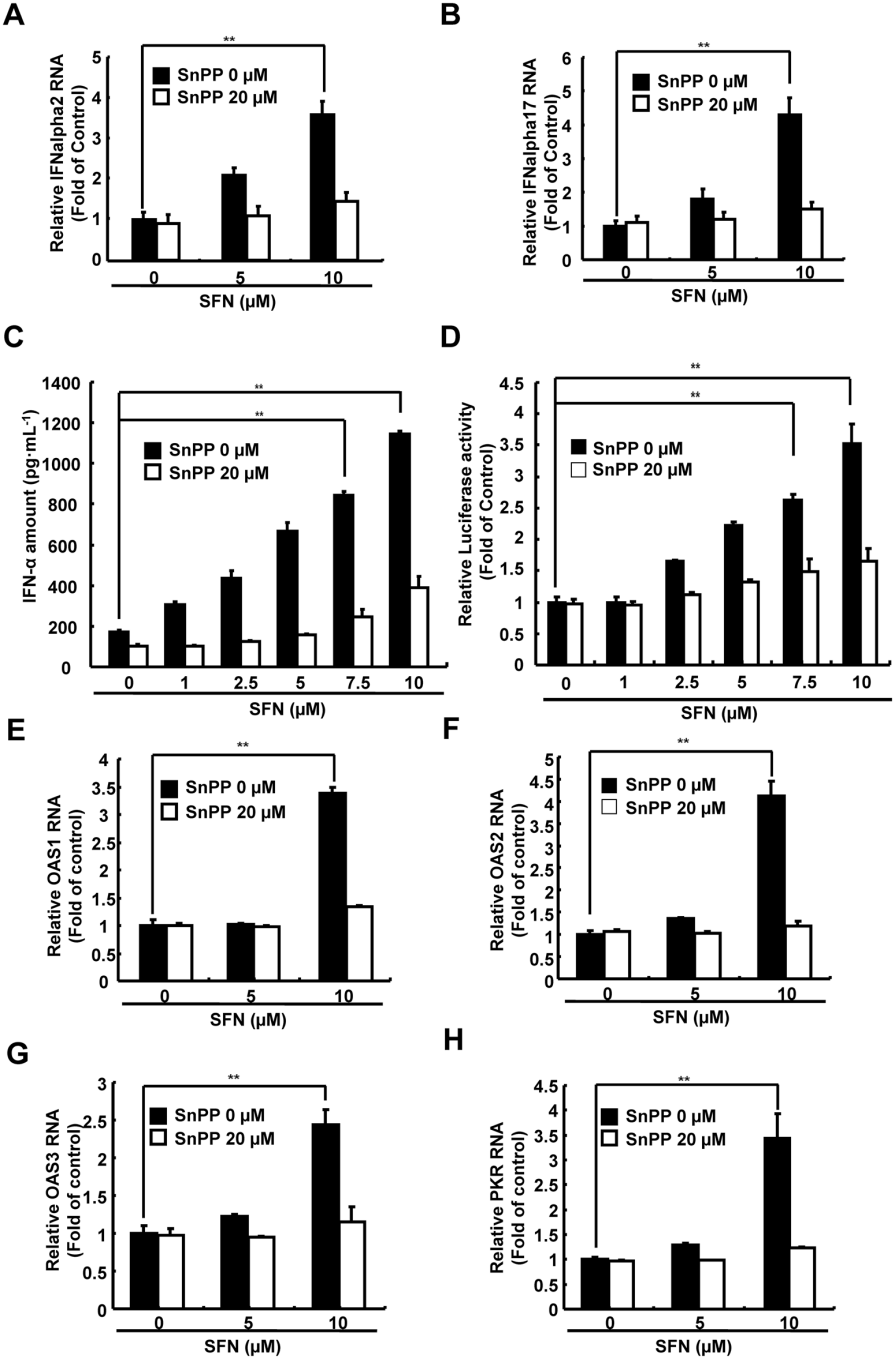
SFN stimulated antiviral IFN response in HCV replicon system. (A and B) SFN induced expression of IFN-α2 and -α17 genes, which was attenuated by SnPP. Ava5 cells were treated with the indicated concentrations of SFN with or without SnPP (20 μM) for 3 days. The relative RNA levels of IFN-α2, and IFN-α17 were analyzed by qRT-PCR following normalization of the internal control *gapdh*. (C) SFN induced IFN-α secretion that was attenuated by SnPP. Ava5 cells were treated with the indicated concentrations of SFN with or without SnPP (20 μM) for 3 days. The supernatants of SFN-treated Ava5 cells were harvested and IFN-α secretion was measured by the IFN ELISA kit. (D) SFN induced ISRE activity that was attenuated by SnPP. The pISRE-Luc- and vehicle-transfected Ava5 cells were treated with the indicated concentrations of SFN (0–10 μM) with or without SnPP (20 μM) for 3 days. The relative ISRE activity was analyzed by the luciferase assay. (E–H) SFN induced OAS gene family and PKR gene expression, which were attenuated by SnPP. Ava5 cells were treated with the indicated concentrations of SFN with or without SnPP (20 μM) for 3 days. The relative RNA levels of OAS1, OAS2, OAS3, and PKR were analyzed by qRT-PCR following normalization of the internal control *gapdh*. The relative luciferase activity and RNA levels of untreated cells were defined as 1. Data were represented as the means of normalized data ± standard deviations (error bars) based on five independent experiments. *P < 0.05; ** P < 0.01.

**Fig 6:**
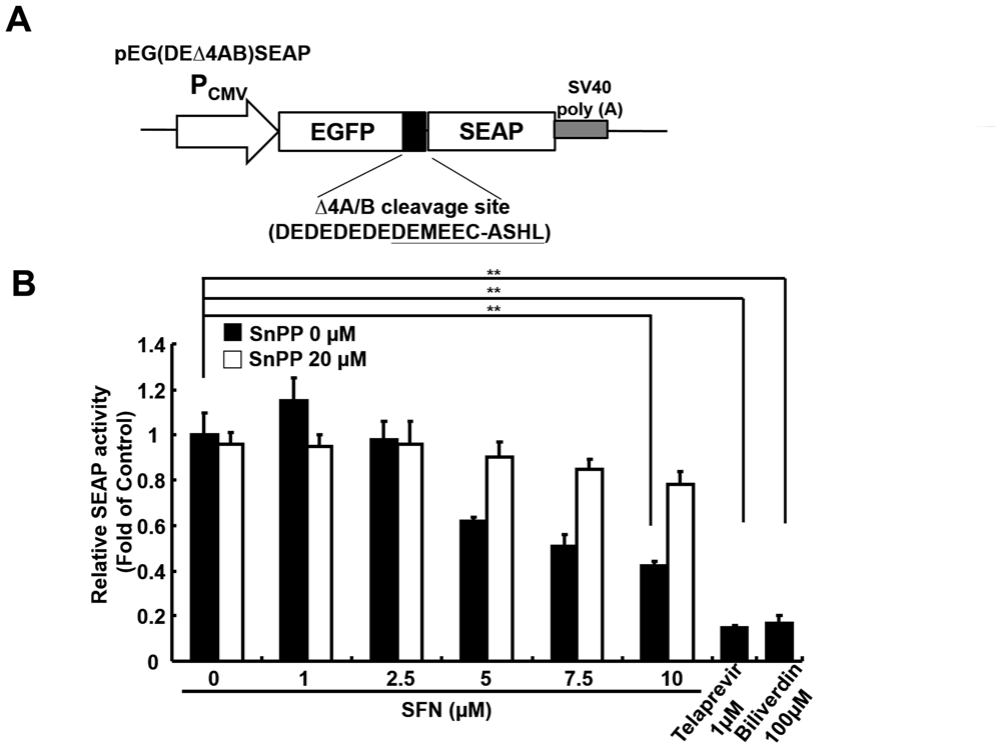
SFN inhibited HCV NS3/4A protease activity. (A) Scheme of a response vector containing an NS3 cleavage site between enhanced green fluorescent protein (EGFP) and secreted alkaline phosphatase (SEAP) (pEGFP-EGΔ4AB-SEAP). (B) HCV protease activity was reduced by SFN treatment in a concentration-dependent manner. The NS3 response reporter vector, pEG(DEΔ4AB)SEAP and the NS3 expression vector pCMV-NS3/4A-myc were cotransfected into Huh-7 cells before treatment with the indicated concentrations of SFN (0–10 μM) with or without SnPP (20 μM) for 3 days. The culture supernatants were harvested and the SEAP activity was analyzed by Phospha-Light assay kit. Telaprevir (1 μM) and biliverdin (100 μM) served as positive controls for anti-HCV protease activity. The relative SEAP activities were presented as fold changes compared to untreated cells, in which as the activity was considered to be 1. Data were represented as the means of normalized data ± standard deviations (error bars) from five independent experiments. *P < 0.05; ** P < 0.*01*.

To determine whether SFN increased the production of biliverdin, an effective HO-1 product against HCV, the Ava5 cells were treated with increasing concentrations of SFN for 3 days. The amount of bilirubin, a biliverdin metabolite, was measured as a surrogate by MeDiPro direct bilirubin test kit. As shown in Fig 4A, SFN led to a significant increase in bilirubin levels in Ava5 cells compared to the basal levels detected in parental Huh-7 cells. Because biliverdin is converted into bilirubin by biliverdin reductase (BVR) [28], we further determined whether SFN influenced BVR expression in SFN-treated Ava5 cells by qRT-PCR and Western blotting. As shown in Fig 4B and 4C, we did not detect any significant changes in BVR RNA or protein levels following SFN treatment. Because production between biliverdin and bilirubin is symmetrical, these results suggest that the SFN-mediated changes in biliverdin production occurred through HO-1 induction. To verify whether SFN inhibited HCV replication by HO-1-mediated induction of IFN gene expression, including IFN-α2 and IFN-α17, we assessed the gene expression in SFN-treated Ava5 cells by qRT-PCR. As shown in Fig 5A and 5B, SFN significantly induced the IFN-α2 and IFN-α17 expression in SFN-treated Ava5 cells compare to SFN-untreated controls that were attenuated with SnPP treatment. We further performed an ELISA assay to confirm whether SFN significantly increased IFN-α levels in culture supernatants at effective antiviral concentrations, which were attenuated in SnPP co-treated cultures (Fig 5C). To verify whether SFN inhibited HCV replication by HO-1-mediated induction of antiviral IFN response, we performed a transient ISRE activity assay in Ava5 cells. The reporter plasmid pISRE-Luc was transfected into Ava5 cells that were then treated with increasing SFN concentrations with or without the HO-1 specific inhibitor SnPP for 3 days. As shown in Fig 5D, SFN led to a significant induction of ISRE-mediated luciferase activity at effective antiviral concentrations, which was attenuated with SnPP treatment. Subsequently, antiviral IFN-stimulated genes, including 2′-5′-oligoadenylate synthetase (OAS) 1, OAS2, OAS3, and protein kinase R (PKR), were assessed in SFN-treated Ava5 cells by qRT-PCR. As shown in Fig 5E–5H, SFN significantly induced the expression of all the investigated IFN response genes in SFN-treated Ava5 cells compared to those in untreated controls; however, these inductive effects were attenuated by SnPP treatment. To verify whether SFN inhibited HCV replication through HO-1-mediated inhibition in HCV NS3/4A protease activity, as shown in Fig 6A, we used a trans-reporter system that included an expression plasmid containing NS3/4A (pCMV-NS3/4A-myc) and response reporter pEG(DEΔ4AB)SEAP with a cleavage site of HCV protease for co-transfection into Huh-7 cells. The transfected cells were treated with increasing concentrations of SFN with or without SnPP for 3 days, and the amount of SEAP was used as a readout for HCV protease activity. As shown in Fig 6B, SFN significantly decreased the HCV protease activity compared to untreated controls, which was restored with SnPP treatment. The equal amount of NS3 expression was confirmed by western blotting (S3 Fig). Taken together, these results suggested that SFN-mediated HO-1 induction leads to biliverdin production, which in turn inhibits HCV replication through the induction of antiviral IFN response and the inhibition of anti-HCV protease activity.

### SFN Up-Regulates Nrf2 Transactivating HO-1 Expression to Inhibit HCV Replication

**Fig 7:**
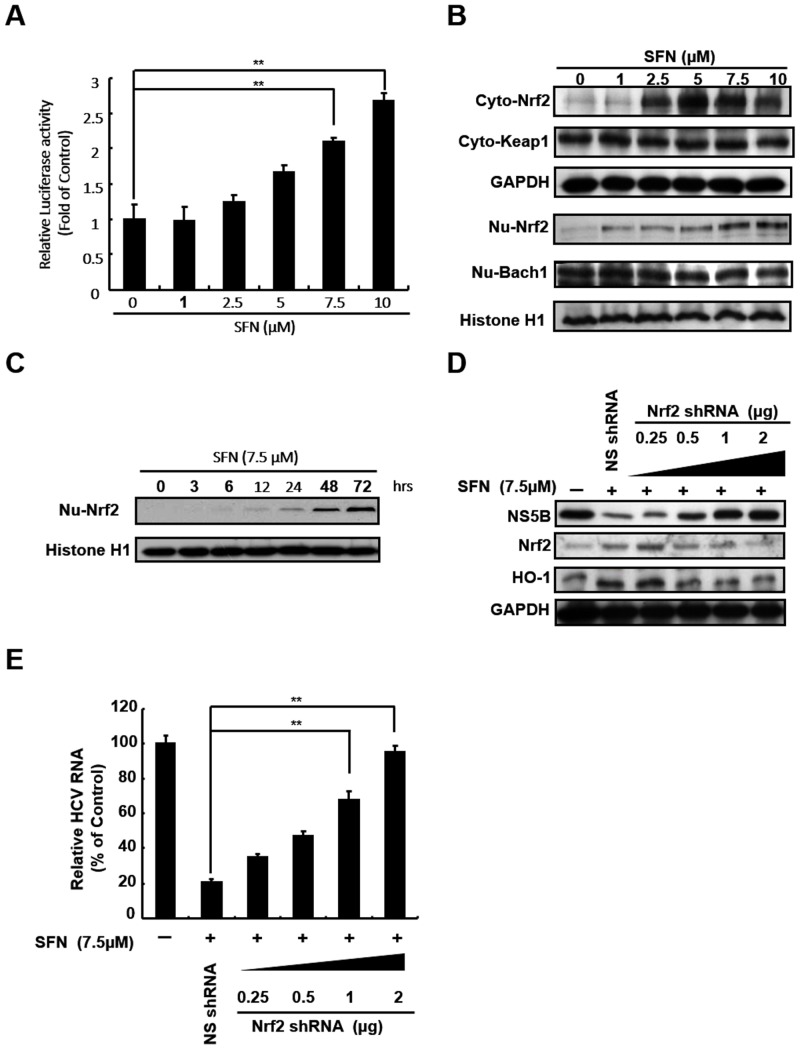
SFN inhibited HCV replication by upregulating Nrf2 expression. SFN stimulated (A) ARE transactivation, (B) Nrf2 expression, and (C) Nrf2 nuclear translocation in a concentration- or time-dependent manner. The antioxidant response reporter plasmid, p3xARE-Luc, was transfected into Ava5 cells and then treated with the indicated concentrations of SFN (0–10 μM) for 3 days. The relative induction of antioxidant activity was determined by luciferase assay. The activity of SFN-untreated Ava5 cells was considered to be 1. The lysates of cytoplasmic and nuclear fractions were separated and the expression levels of HO-1 regulators were analyzed by Western blotting. The SFN-induced Nrf2 nuclear translocation was analyzed by Western blotting at different incubation times (0–72 h). (D and E) Anti-HCV activity of SFN was attenuated by Nrf2 shRNA against Nrf2 expression. Increasing amounts of Nrf2-specific shRNA (0.25–2 μg) or non-specific shRNA were transfected into Ava5 cells. The transfected cells were then treated with 7.5 μM SFN for 3 days. HCV and HO-1 protein and RNA levels were analyzed by Western blotting and qRT-PCR, respectively. Western blotting was performed using antibodies against HCV NS5B, Nrf2, Keap1, Bach1, and HO-1. The expression levels of GAPDH and Histone 1 showed equal loading of cell lysates. Relative RNA levels were normalized to the internal control *gapdh*. The relative HCV RNA levels were presented as percentage changes compared to SFN-untreated/untransfected Ava5 cells, in which the level was considered to be 100%. Data were represented as the means of normalized data ± standard deviations (error bars) based on five independent experiments. *P < 0.05; ** P < 0.*01*.

HO-1 expression is regulated by the transcription factors Nrf2, Keap1, and Bach1 through the binding of ARE in its promoter region [29, 30]. To determine whether SFN-mediated HO-1 induction was dependent on ARE transactivation, the p3xARE-Luc-transfected Ava5 cells were treated with increasing concentrations of SFN for 3 days. As shown in Fig 7A, at effective antiviral concentrations, SFN significantly increased the ARE-mediated luciferase activity. Therefore, we determined the effect of SFN on the expression and nuclear translocation of transcription factors regulating HO-1. As shown in Fig 7B, SFN led to a significant increase in the expression of Nrf2 in both cytoplasmic and nuclear fractions in a concentration-dependent manner. However, we did not observe significant changes in the protein levels of Keap1 and Bach1 under the same experimental conditions. In addition, SFN significantly induced the nuclear accumulation of Nrf2 in a time-dependent manner (Fig 7C). To further assess whether the anti-HCV activity of SFN was dependent on Nrf2-mediated HO-1 induction, Ava5 cells were transfected with increasing concentration of Nrf2 shRNA before being treated with 7.5 μM SFN for 3 days. HCV protein synthesis and RNA replication were analyzed by Western blotting and qRT-PCR, respectively. As shown in Fig 7D, the reduction in HCV protein synthesis by SFN treatment was restored with Nrf2 knockdown, which led to a decrease in HO-1 expression. Similar to western blotting results, Nrf2 knockdown attenuated SFN-reduced HCV RNA levels in a dose-dependent manner (Fig 7E). Taken together, these results suggested that the anti-HCV activity of SFN was dependent on Nrf2-mediated HO-1 induction.

### SFN Stimulates PI3K Phosphorylation Contributing to Nrf2/HO-1-Mediated Inhibition of HCV Replication

**Fig 8:**
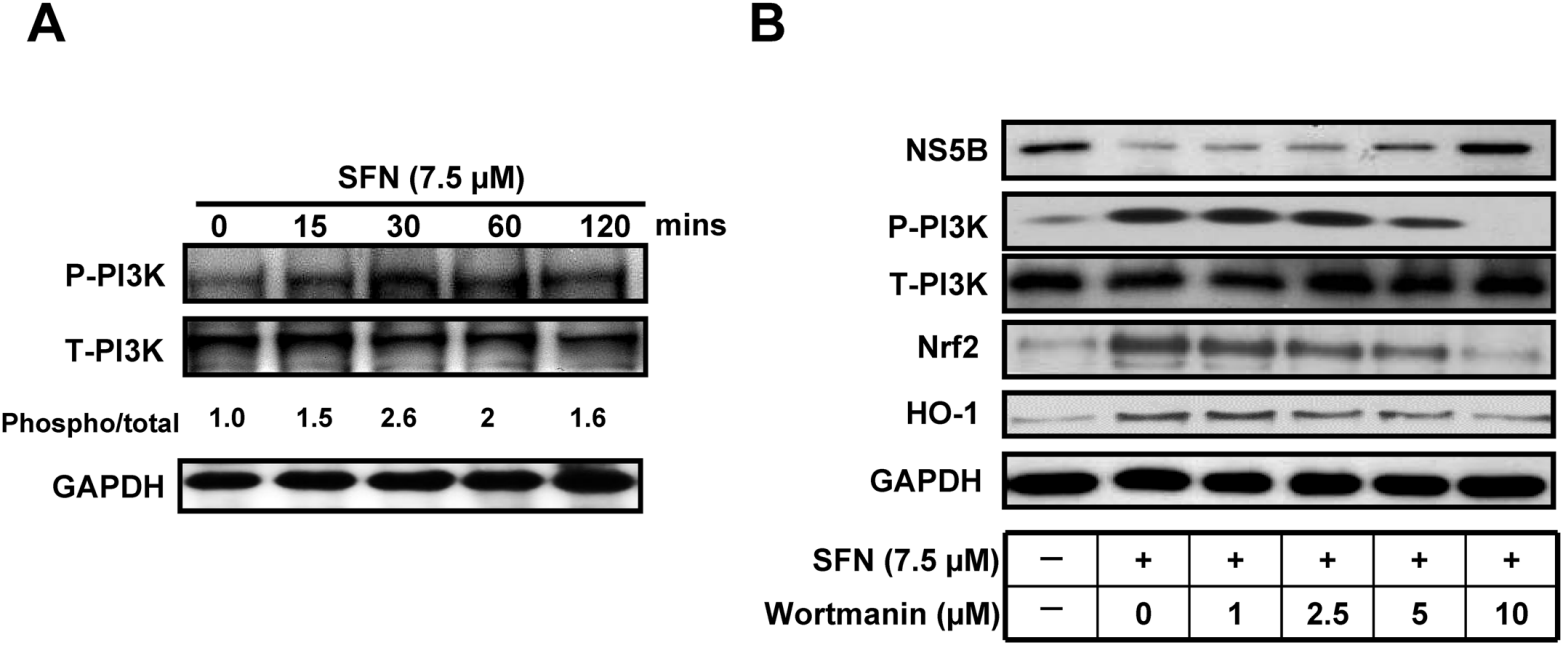
SFN-mediated Nrf2/HO-1 activation was PI3K dependent. (A) Phosphorylation of PI3K was induced by SFN treatment. Ava5 cells were treated with 7.5 μM SFN, the cellular lysates were collected at the indicated time points (0–120 min), and the phosphorylation level of PI3K was analyzed by Western blotting. (B) Anti-HCV activity of SFN was attenuated by PI3K inhibition. Ava5 cells were treated with 7.5 μM SFN with or without the PI3K-specific inhibitor, wortmanin (0–10 μM), for 3 days. The HCV protein, Nrf2, and HO-1 expression were analyzed by Western blotting. Western blotting that was performed using antibodies against phospho-PI3K, total-PI3K, HCV NS5B, Nrf2, and HO-1. An antibody against GAPDH was used to show equal loading of lysates.

**Fig 9:**
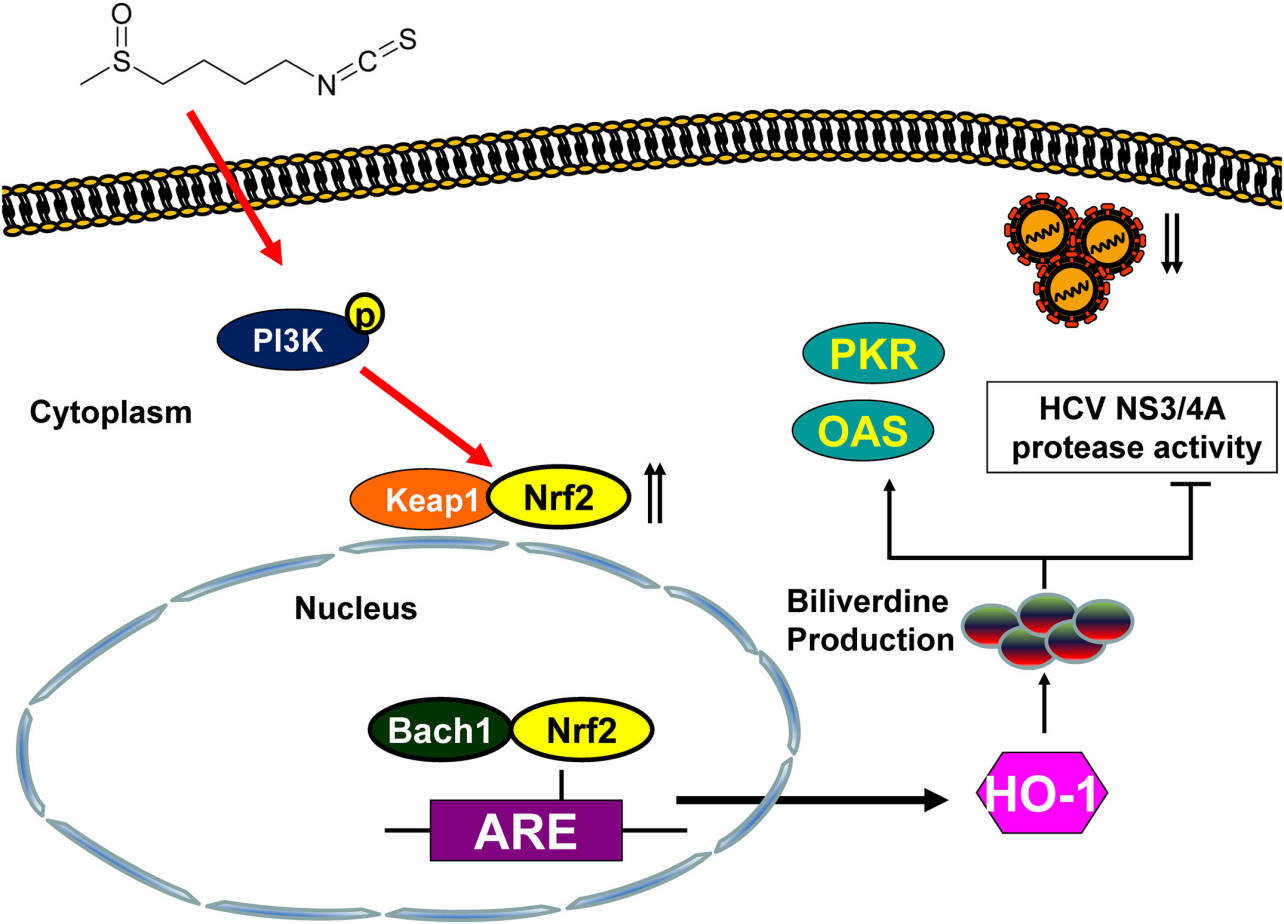
Scheme of SFN-mediated inhibition of HCV replication. SFN induces PI3K phosphorylation and subsequently triggers Nrf2 nuclear translocation to activate HO-1 expression leading to biliverdin production against HCV replication by the induction of antiviral IFN responses and suppression of HCV NS3/4A protease activity.

Numerous studies have shown that SFN induces HO-1 expression through the activation of PI3K/Akt signaling cascade [31, 32]. To investigate the role of PI3K in the anti-HCV activity of SFN, we determined Whether SFN induced PI3K phosphorylation in Ava5 cells that were treated with 7.5 μM SFN by western blotting at the indicated time points shown in Fig 8A. SFN significantly induced the phosphorylation of PI3K 15 min after treatment. Furthermore, we treated SFN-treated Ava5 cells with increasing concentrations of a PI3K specific inhibitor, wortmannin, to clarify the role of PI3K in the anti-HCV activity of SFN. The protein levels of Nrf2, HO-1, and HCV were analyzed by Western blotting, and as shown in Fig 8B, the inhibition of HCV protein synthesis by SFN treatment was attenuated by PI3K inhibitor treatment. In parallel, as expected, SFN-induced Nrf2 and HO-1 levels were attenuated with the PI3K inhibitor treatment. Taken together, these results indicated that SFN inhibited HCV replication through Nrf2-mediated HO-1 induction via the activation of the PI3K signaling pathway (Fig 9).

## Discussion

Oxidative stress is an important pathophysiological mechanism in the progression of HCV-associated liver disease. Accumulating evidence suggests a close correlation between HCV infection and oxidative stress and that chronic liver damage caused by viral infection and replication correlates with ROS accumulation[33]. HCV core and NS5A protein are shown to increase ROS formation in multiple mechanisms [34, 35]. These findings indicate the potential beneficial effects of antioxidant therapy in HCV treatment. Earlier studies, including a study by our group, have shown that HO-1 induction was associated with anti-inflammatory, antioxidant, and anti-HCV activities [24, 36, 37]. Previous reports demonstrated that SFN can modulates influenza A virus entry and replication in nasal epithelial cells through Nrf2 activation[7]. It is still not confirmed whether this antiviral effect of SFN is due to Nrf2-mediated HO-1 induction. Here, we clearly revealed that SFN significantly inhibited HCV replication through HO-1 induction and that Nrf2-medated HO-1 induction was correlated with the antiviral activity of SFN (Figs 3, 5, and 6). We believe that SFN may serve as an antiviral agent or a potential lead compound for the development of new antiviral therapeutics.

HCV infection and protein expression are known to induce pro-oxidant activity, cause glutathione depletion, and alter the expression of oxidative defense enzymes *in vivo* and *in vitro*, including HO-1 [38, 39]. In addition, in contrast to our findings, other groups found significant induction of HO-1 expression in replicon cells [40–42]. We suggested that the differences between each replicon system may be contributed by the reasons of selective subclones or time of culture subpassage, eventually leading to distinct cellular responses. However, Abdalla et al. demonstrated that HO-1 expression was reduced in HCV patients and with core protein overexpression [38]. They suggested that HCV-reduced HO-1 expression would lead to the impairment of host response against oxidative stress for the progression of HCV-associated chronic liver disease. Additionally, increases in the levels of proinflammatory mediators, such as tumor necrosis factor (TNF)-α, inducible nitric oxide synthase (iNOS), cyclooxygenase-2 (COX-2), and interleukin-1(IL-1), caused by HCV infection are known to increase the risk of HCC [43]. More recently, anti-inflammatory activity of HO-1 has been proposed to be a promising theraputic strategy against gastrointestinal diseases, including gastrointestinal tract, liver, and pancreas [44]. In addition, various phytocompounds display their bioactivity as anti-inflammatory and anti-carcinogenic molecules through the induction of Nrf2/HO-1 pathway [36, 45–48]. Therefore, the modulation of HO-1 by SFN activation may contribute to hepatic protection, reinforcing SFN as a potential hepatoprotective agent with dual actions: elimination of HCV infection and prevention of HCC development. In addition, in human HepG2 cells, SFN induced apoptosis at higher concentrations and antioxidant defenses via Nrf2-mediated gene regulation at low concentrations [46]. Here, we found that SFN-mediated increases in HO-1 promoter activity, mRNA, and protein levels were dose-dependent and associated with Nrf2 activation (Fig 7). Taken together, these findings indicates that SFN regulates the antioxidant defenses against HCV replication.

During the past few years, several direct-acting antivirals (DAAs) targeting viral protein have been successfully developed against HCV infection [49]. However, the therapeutic effects of these DAAs are limited because of variations in the sensitivity of different HCV genotypes and spontaneous viral mutations. Cellular proteins influencing viral infections are being considered as promising candidates to prevent viral resistance and increase drug susceptibility because the mutation rate of host genome is lower than that of viral RNA genome [50]. Natural products have served as an important source for drug development in the last decade, with more than 50% of plant-derived compounds being used as pharmaceuticals in clinical practice [51]. Because several cellular factors can be modulated using phytochemicals, many plant-derived compounds show significant cytoprotective effects in various diseases [52]. In the field of HCV research, several plant-derived antioxidant compounds, such as curcumin, silymarin, silibinin, and epigallocatechin-3-gallate (EGCG) have shown significant antiviral activity in different stages of the HCV life cycle [53–57]. In addition, several natural products with significant antioxidant activity also induce HO-1 against HCV replication, such as lucidone, andrographolide, and curcumin [24, 25, 53]. However, each of compound induces HO-1 expression through different molecular mechanism and has distinct chemical formula. Furthermore, there are many regulation factors involved in HO-1 induction, including NF-kB, AP-1, p38, PKC, PI3K/AKT, glycogen synthase kinase 3 beta, and JAK/STAT signaling pathways [58]. In the present study, we found that the anti-HCV activity of SFN was dependent on PI3K activation, which is consistent with the results of up-regulation of Nrf2-meidated HO-1 expression by geniposide and punicalagin [59, 60]. However, studies are still needed to further elucidate any additional molecular pathways involved in the anti-HCV activity of SFN by induction of p38 MAPK, suppression of NF-κB-mediated COX-2, or modulation of ROS production [19, 25, 31]. Consequently, plants are potential sources of new bioactive compounds against HCV infection. We concluded that HO-1 should be considered as an important antiviral factor in HCV replication.

Here we demonstrated that SFN possesses effective anti-HCV activity and further identified the PI3K/Nrf2 molecular pathway of HO-1 induction (Fig 9). In addition, SFN, in combination with recently US FDA-approved drugs, showed significant synergistic effects on HCV replication *in vitro* (Tables 3 and 4). Furthermore, considering cruciferous vegetables to be popular dietary supplements, SFN may be a desirable antiviral therapeutic agent with minimal side effects for HCV patients. In previous studies, SFN has been shown chemopreventive effects on chemical-induced chemically-induced breast, colon and stomach cancers in rats [61–64]. In addition, the pharmacokinetic studies of SFN have performed in animal and human studies. The results shows that SFN metabolites significant increase in concentration- and time-dependent manner in liver [65, 66]. Hu et al. also demonstrated that the plasma concentration of SFN occurred at 1 h and reached around 20 μM within 4h in rats after oral dosing of 50 μmol of SFN. SFN displayed rapid absorption and the plasma concentration declined with an elimination half-life of 2.2h [67]. However, further investigation to confirm the effectiveness of SFN-mediated antiviral activity *in vivo* is necessary. Taken together, our results reveal the beneficial effects of SFN in anti-HCV replication and suggest that SFN is useful for development of new therapeutics for HCV infection and HCV-associated liver diseases.

## Supporting Information

S1 Fig SFN suppressed HCV replication in the JFH-1 replicon system. Huh7.5/J6/JFHEMCVIRESRlucNeo cells were exposed to the indicated SFN concentrations (0–10 μM) for 3 days. The Renilla luciferase activities are used to determine the level of replication efficiency. The relative viral replication efficiency was presented as percentage changes compared to the SFN-untreated cells, which were considered as 100%. Data were presented as the means of normalized data ± standard deviations (error bars) based on five independent experiments. *P < 0.05; ** P < 0.01.
(PDF)

S2 Fig SFN had no significant effect on HCV viral entry and assembly. (A) Huh7.5 cells were infected with JFH-1 and treated with SFN for 1 hr at 37°C and then the infectecells were washed and incubated in complete culture medium. The intracellular HCV RNA copy numbers were quantified by qRT-PCR. The treatment of heparin served as positive control for the inhibition of HCV entry. The relative HCV entry were presented as percentage changes compared to the SFN-untreated cells, which were considered as 100%. (B) Huh7.5 cells were infected with JFH-1 and then treated with SFN for 72 hrs. The supernatant and cell lysate were collected and the HCV RNA copy numbers were quantified by qRT-PCR. Data were represented as the means of normalized data ± standard deviations (error bars) based on five independent experiments. *P < 0.05; ** P < 0.01.
(PDF)

S3 Fig The equal amount of HCV NS3 expression in protease reporter system. The NS3 response reporter vector, pEG(DEΔ4AB)SEAP and the NS3 expression vector pCMV-NS3/4A-myc were cotransfected into Huh-7 cells before treatment with the indicated concentrations of SFN (0–10 μM) with or without SnPP (20 μM) for 3 days. HCV NS3 protein expression were analyzed by Western blotting with specific antibody against GAPDH and HCV NS3. GAPDH expression was used as the protein loading control.
(PDF)
