# Supplementary figures and images for: Sulforaphane Suppresses Hepatitis C Virus Replication by Up-Regulating Heme Oxygenase-1 Expression through PI3K/Nrf2 Pathway
Source: PLoS One. 2016 Mar 29;11(3):e0152236. doi: 10.1371/journal.pone.0152236 (PMC4811417; doi:10.1371/journal.pone.0152236)

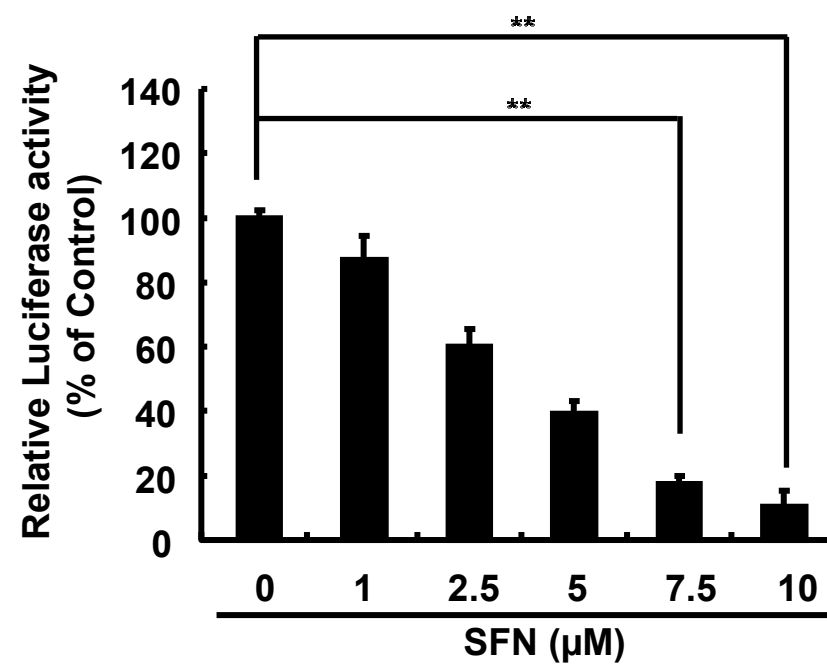

Supplement: S1 Fig — Huh7.5/J6/JFHEMCVIRESRlucNeo cells were exposed to the indicated SFN concentrations (0–10 μM) for 3 days. The Renilla luciferase activities are used to determine the level of replication efficiency. The relative viral replication efficiency was presented as percentage changes compared to the SFN-untreated cells, which were considered as 100%. Data were presented as the means of normalized data ± standard deviations (error bars) based on five independent experiments. *P < 0.05; ** P < 0.01. (PDF) [file pone.0152236.s001.pdf]

**A**

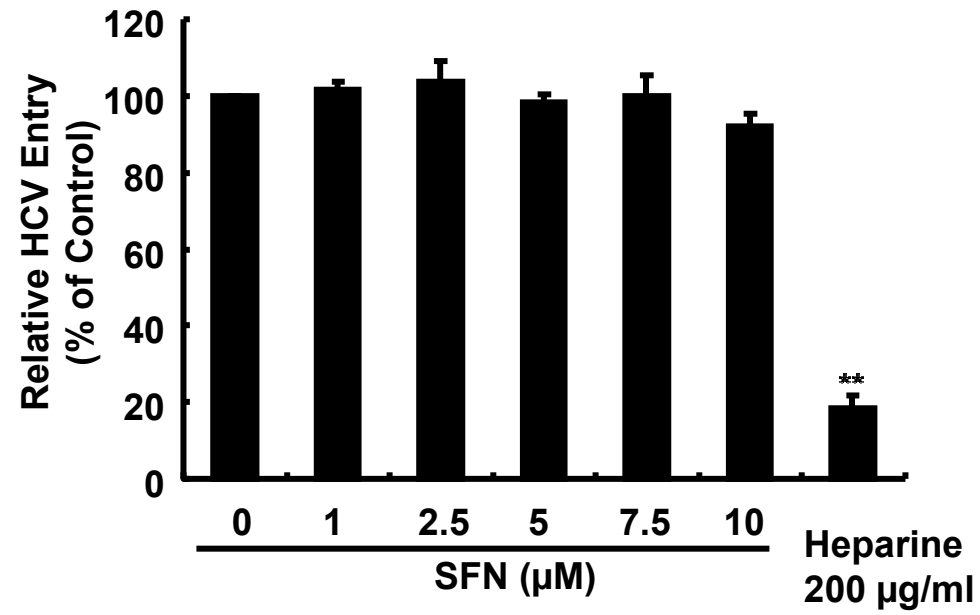

**B**

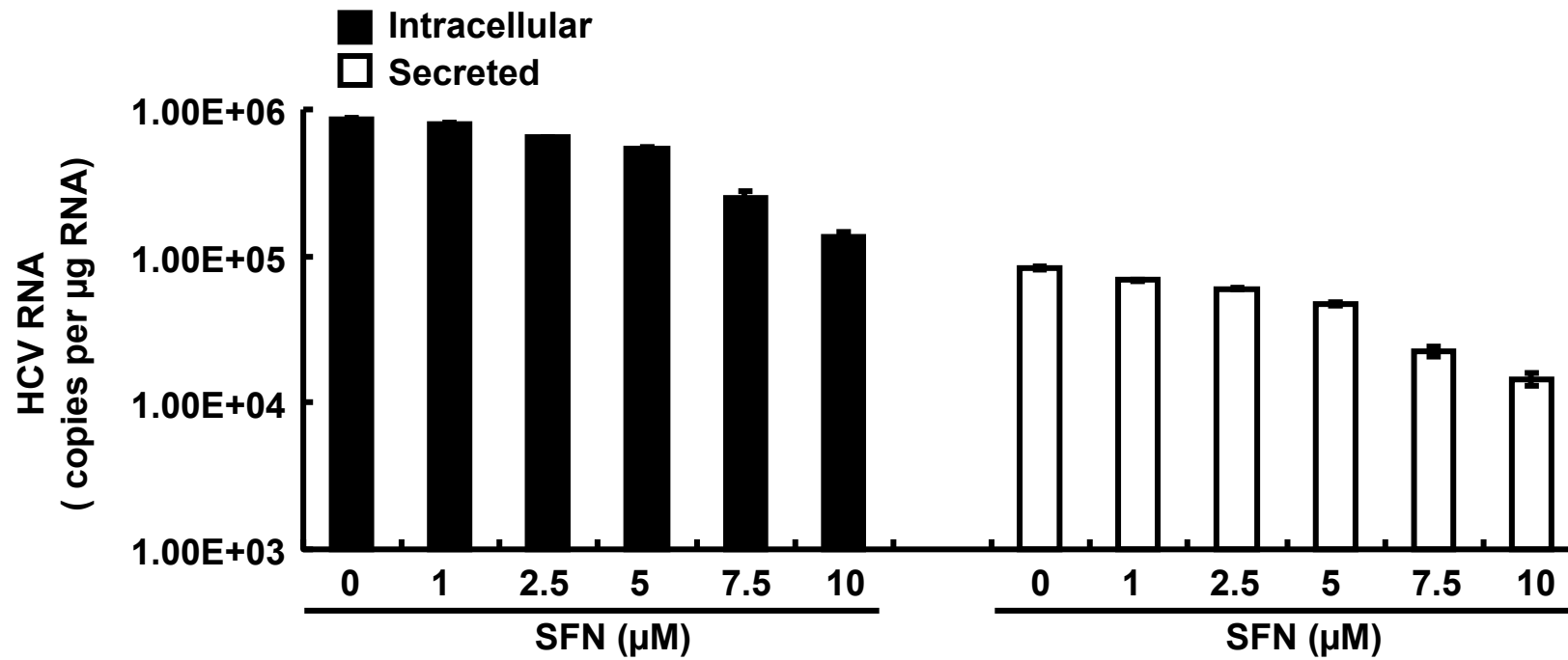

Supplement: S2 Fig — (A) Huh7.5 cells were infected with JFH-1 and treated with SFN for 1 hr at 37°C and then the infectecells were washed and incubated in complete culture medium. The intracellular HCV RNA copy numbers were quantified by qRT-PCR. The treatment of heparin served as positive control for the inhibition of HCV entry. The relative HCV entry were presented as percentage changes compared to the SFN-untreated cells, which were considered as 100%. (B) Huh7.5 cells were infected with JFH-1 and then treated with SFN for 72 hrs. The supernatant and cell lysate were collected and the HCV RNA copy numbers were quantified by qRT-PCR. Data were represented as the means of normalized data ± standard deviations (error bars) based on five independent experiments. *P < 0.05; ** P < 0.01. (PDF) [file pone.0152236.s002.pdf]

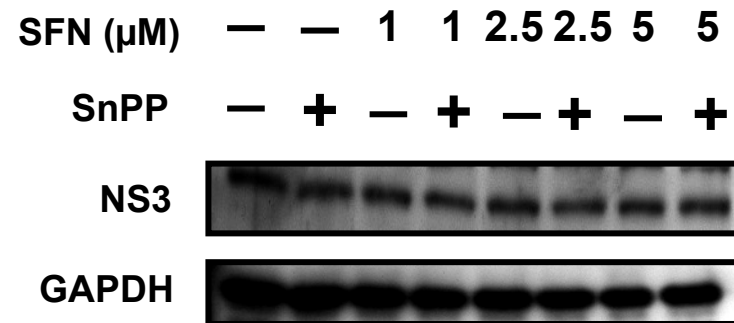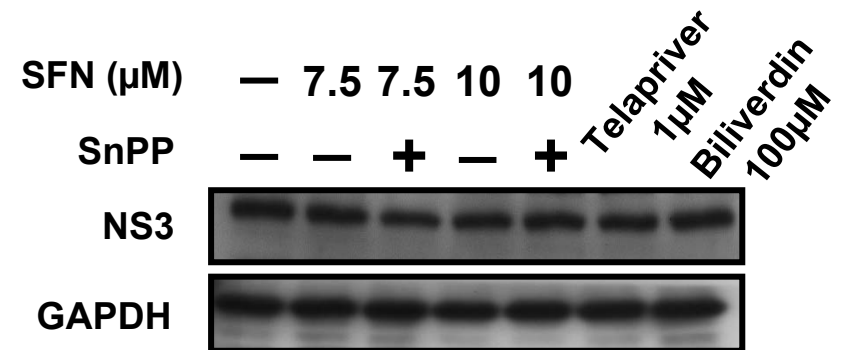

Supplement: S3 Fig — The NS3 response reporter vector, pEG(DEΔ4AB)SEAP and the NS3 expression vector pCMV-NS3/4A-myc were cotransfected into Huh-7 cells before treatment with the indicated concentrations of SFN (0–10 μM) with or without SnPP (20 μM) for 3 days. HCV NS3 protein expression were analyzed by Western blotting with specific antibody against GAPDH and HCV NS3. GAPDH expression was used as the protein loading control. (PDF) [file pone.0152236.s003.pdf]
